# A Meta-Learning-Based Ensemble Model for Explainable Alzheimer’s Disease Diagnosis

**DOI:** 10.3390/diagnostics15131642

**Published:** 2025-06-27

**Authors:** Fatima Hasan Al-bakri, Wan Mohd Yaakob Wan Bejuri, Mohamed Nasser Al-Andoli, Raja Rina Raja Ikram, Hui Min Khor, Zulkifli Tahir, The Alzheimer’s Disease Neuroimaging Initiative

**Affiliations:** 1 Faculty of Information and Communication Technology, Universiti Teknikal Malaysia Melaka, Melaka 76100, Malaysia; forignn@gmail.com (F.H.A.-b.); raja.rina@utem.edu.my (R.R.R.I.); 2 Faculty of Artificial Intelligence and Cyber Security, Universiti Teknikal Malaysia Melaka, Melaka 76100, Malaysia; 3 Faculty of Computing Informatics, Multimedia University, Cyberjaya 63100, Malaysia; 4 Department of Medicine, Faculty of Medicine, University of Malaya, Kuala Lumpur 50603, Malaysia; hmkhor@um.edu.my; 5 Faculty of Engineering, Universitas Hasanuddin, Gowa 92171, Indonesia; zulkifli@unhas.ac.id

**Keywords:** Alzheimer’s disease diagnosis, explainable artificial intelligence (XAI), ensemble learning, mid-slice MRI, lateral ventricles, clinical data, MMSE, interpretable models, neuroimaging, meta-model

## Abstract

**Background/Objectives:** Artificial intelligence (AI) models for Alzheimer’s disease (AD) diagnosis often face the challenge of limited explainability, hindering their clinical adoption. Previous studies have relied on full-scale MRI, which increases unnecessary features, creating a “black-box” problem in current XAI models. **Methods:** This study proposes an explainable ensemble-based diagnostic framework trained on both clinical data and mid-slice axial MRI from the ADNI and OASIS datasets. The methodology involves training an ensemble model that integrates Random Forest, Support Vector Machine, XGBoost, and Gradient Boosting classifiers, with meta-logistic regression used for the final decision. The core contribution lies in the exclusive use of mid-slice MRI images, which highlight the lateral ventricles, thus improving the transparency and clinical relevance of the decision-making process. Our mid-slice approach minimizes unnecessary features and enhances model explainability by design. **Results:** We achieved state-of-the-art diagnostic accuracy: 99% on OASIS and 97.61% on ADNI using clinical data alone; 99.38% on OASIS and 98.62% on ADNI using only mid-slice MRI; and 99% accuracy when combining both modalities. The findings demonstrated significant progress in diagnostic transparency, as the algorithm consistently linked predictions to observed structural changes in the dilated lateral ventricles of the brain, which serve as a clinically reliable biomarker for AD and can be easily verified by medical professionals. **Conclusions:** This research presents a step toward more transparent AI-driven diagnostics, bridging the gap between accuracy and explainability in XAI.

## 1. Introduction

Alzheimer’s disease (AD) is a progressive neurodegenerative disorder that affects millions of people worldwide, leading to cognitive decline and memory loss [1]. Early and accurate diagnosis is essential for effective intervention and patient care [2].

AI contributes significantly to the advanced digital age, impacting various human interactions and decision-making processes [3]. AI plays a crucial role in advancing the diagnosis of AD, as the field of diagnosis is one of the most prominent applications of AI in medicine. However, no real translation into clinical practice has been achieved due to the difficulty of interpreting AI decision-making processes in diagnosis, rendering them black-box models for medical practitioners [4].

Recent advancements in XAI have focused on improving explainability while maintaining high diagnostic performance. One key approach involves hybrid interpretation methods, which combine model-agnostic and model-specific techniques to enhance explanation reliability [5]. Additionally, integrating domain knowledge into explanation frameworks has been explored to improve the clinical relevance of AI-driven insights [6].

Another critical development is the enhancement of clinical relevance by focusing on anatomically meaningful regions, which is a well-established biomarker for AD [7]. Researchers have also incorporated established biomarkers into AI-generated explanations, further aligning model outputs with existing medical knowledge [8]. Moreover, a user-centered design approach has been emphasized to bridge the gap between AI models and clinical applications. This includes the development of clinician-friendly explanation interfaces that present model decisions in an intuitive manner [9].

Additionally, the validation of AI-generated explanations through medical professional assessments has been proposed to ensure that these explanations align with real-world diagnostic workflows [10]. These advancements contribute to making AI-driven diagnostic models more interpretable and applicable in clinical settings.

This research aims to develop an explainable, AI-based diagnostic tool for AD, focusing on relevant clinical features. Unlike traditional models that process complete MRI scans, our approach targets the middle slice of the brain, where key structures such as the lateral ventricles are visible, to reduce computational complexity and enhance explainability. By integrating imaging data with clinical features, the proposed model aims to provide reliable predictions and interpretations consistent with known medical biomarkers, making it more accessible and useful for GPs in real-world clinical settings. The key contributions of this work are as follows:

1. Enhanced Transparency: To address the improving model explainability, our approach simplifies the input space by training exclusively on mid-slice MRI scans. These slices highlight the lateral ventricles, facilitating the explanation of AI decisions without compromising accuracy.
2. High Diagnostic Performance: In line with achieving clinically reliable performance, our ensemble model achieves 99% accuracy on OASIS and 97.61% on ADNI when using clinical data alone, and 99.38% (OASIS) and 98.62% (ADNI) with mid-slice MRI. This confirms the robustness of the model and its generalizability across datasets and input methods.
3. Clinical Application: To support the development of a tool suitable for practical clinical use, the model is designed to assist general practitioners and non-specialists in diagnosing AD, particularly in resource-limited settings where access to neurological expertise is scarce. The simplicity and interpretability of the inputs make it deployable in real-world scenarios.

The study was conducted using publicly available data from the ADNI and OASIS databases, which include MRI scans and clinical data. MRI scans were acquired using various scanners, as documented in the original datasets. Data preprocessing, model training, and interpretability analysis were performed using Python (v3.10, Python Software Foundation, Wilmington, DE, USA).

## 2. Background of XAI in Alzheimer’s Diagnosis

In recent years, AI has become a powerful tool in diagnosing AD through analyzing brain images and patient data. Exploring accurate AI approaches is crucial for medical diagnosis, as physicians in busy clinical settings have limited time for each case [11]. AI tools aim to assist general practitioners and non-specialists in diagnosing AD [12,13], especially in the absence of specialists [14]. AI systems can help non-specialist doctors, such as general practitioners or physicians, identify early signs of AD through interpretable output. In clinical settings with high population density or limited resources, where access to neurologists or radiologists may be limited, these tools can support accurate and timely decision-making. This reduces diagnostic delays, eases the workload on specialists, and helps ensure patients are appropriately referred to and treated early in the disease process, improving long-term clinical outcomes.

AI models have demonstrated promising capabilities in detecting AD using clinical data, cognitive assessments, and neuroimaging [15]. But despite their high accuracy, most AI models are considered a “black box”, providing results without explaining the reasons that led to them, which reduces the confidence of doctors in relying on them [16].

**Figure 1:**
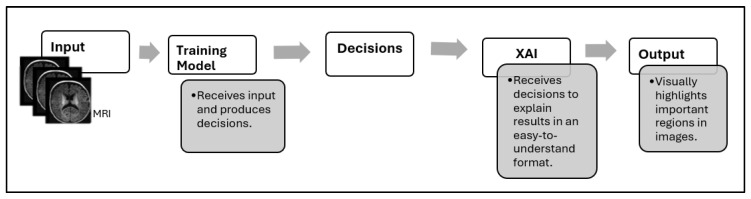
Decision-making workflow with XAI integration.

XAI has emerged as a solution to improve model transparency, which aims to explain how and why a system made its decisions, as shown in Figure 1. But many existing methods still lack practical explainability for clinical applications [5,17,18].

Efforts to improve explainability have led to the development of various explanation techniques, yet these approaches have notable limitations. Post hoc methods, such as SHAP and LIME, provide insights into model behavior but often yield only partial explanations [19]. Model-specific approaches like Grad-CAM offer visual heatmaps to indicate important regions, but they frequently lack clinical context, making their interpretations less meaningful for practitioners [20].

In this research, we developed an AI model based on reducing unnecessary features to enhance the model’s explainability. This makes our model amenable to two explainability techniques: (1) SHAP to explain how clinical factors influence the diagnosis; and (2) Grad-CAM to visualize the important regions in the brain MRI that helped the model make its decision, making it easier for clinicians to validate the model.

## 3. Problem Formulation

While previous research [19,20,21,22,23,24,25,26] has significantly improved the accuracy of AD detection, several critical gaps remain. Almost all existing studies process entire MRI volumes, which increases computational complexity and introduces unnecessary variability in the learning process. Considering the entire scan, these models may capture irrelevant features, increase model complexity, and exacerbate the “black box” problem, making explanation more challenging.

Another key limitation is the inadequate clinical integration of AI models. Many explainability methods [22,24,25,27,28,29] fail to align with established clinical markers, reducing their practical utility in real-world diagnosis, since most studies rely on only one type of data, as shown in Table 1, which makes the explanation not entirely reliable.

Additionally, most existing models are designed for expert radiologists or AI researchers, making them less accessible for non-specialist practitioners who play a crucial role in early diagnosis and patient management [35].

## 4. Our Proposed Approach

Our study proposes a new approach to enhance explainability by training the model exclusively on clinical data and mid-slice axial MRI. This selection translates into two gains: reduced preprocessing and storage costs, and simplified computations during training and inference, as the model processes a focused set of high-diagnostic-value slices rather than the entire 3D MRI volumes.

### 4.1. MRI Slice Selection Strategy

**Figure 2:**
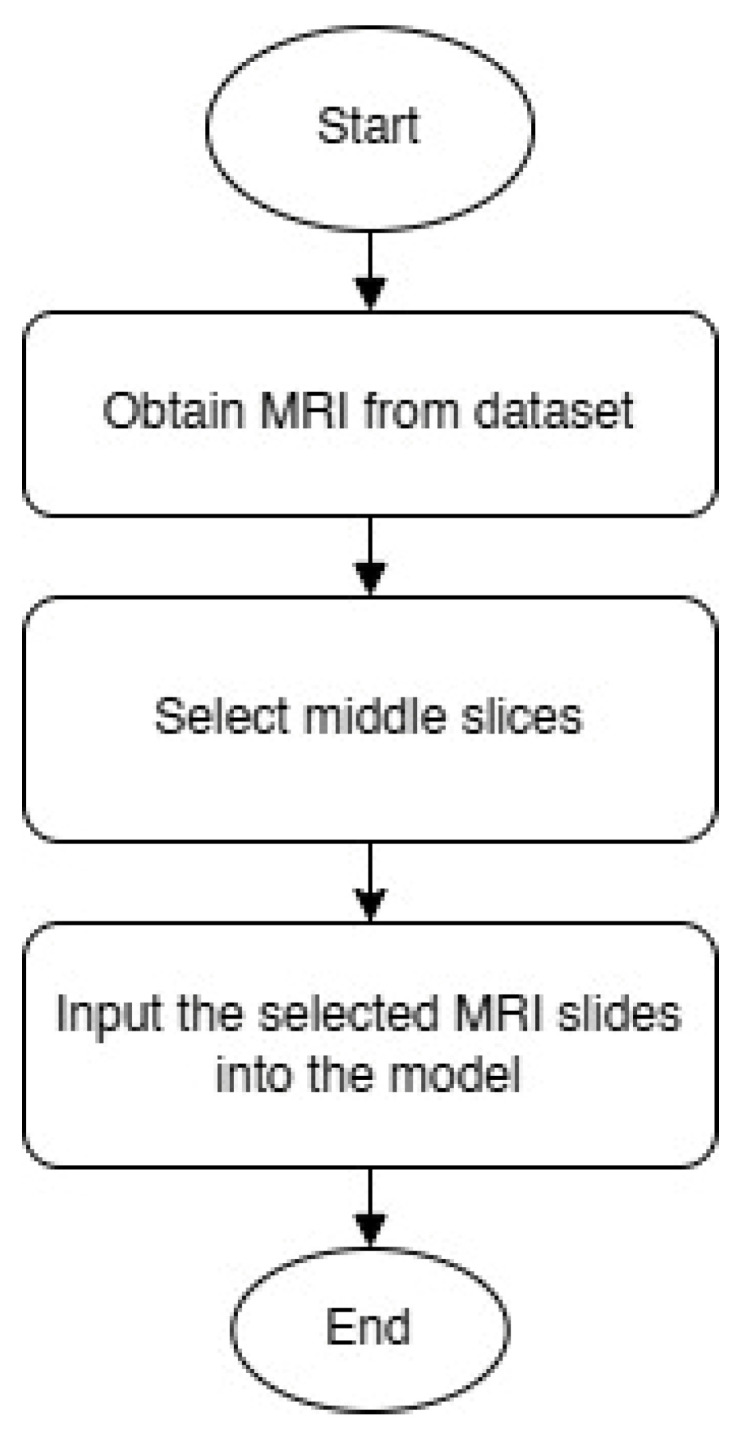
MRI slice selection process.

While previous XAI-focused reviews [36] highlight the role of interpretability in AD classification using MRI and focus on brain regions, none of them provide a clear and logical explanation of how the final decision is made based on the selected image regions. Furthermore, many current AI methods (such as LIME and SHAP applied to whole-slice data or 3D MRI data) tend to highlight areas outside the brain or anatomically irrelevant regions as most important for classification. This raises concerns about the reliability and clinical interpretability of these interpretations. This highlights a gap that our study fills by empirically validating the selection of mid-slice axial MRI and simplifying the interpretability of the decision-making process. Figure 2 shows a flowchart illustrating how diagnostically relevant mid-slices are selected for model input.

We selected only the middle slices of the brain MRI scans, specifically slices that clearly show the lateral ventricles and medial temporal regions. These anatomical structures are known to be among the earliest areas affected by AD [7] and are commonly used in clinical assessments. The mid-slice axial MRI was adopted in the study for two main reasons: (1) its clinical value and (2) the ease of analyzing its results.

**Figure 3:**
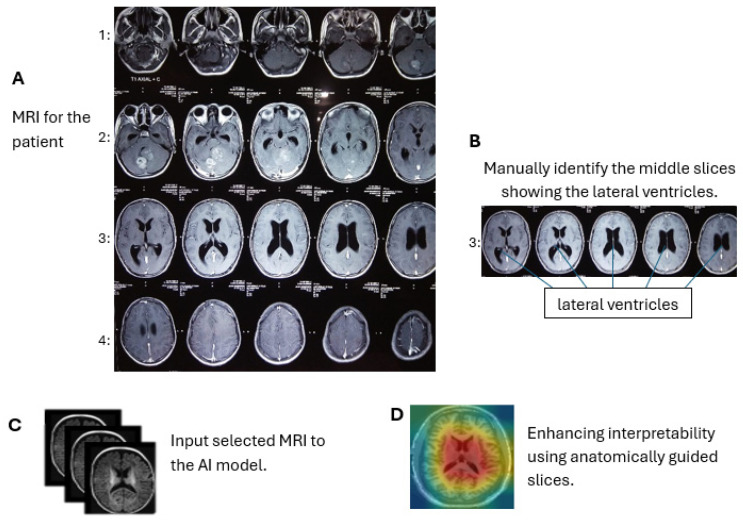
Axial brain MRI at the ventricle level. We use only these mid-slice slides instead of full MRI scans to improve explainability and reduce the “black box” nature of AI models. (**A**) MRI visualization, (**B**) mid-slice selection, (**C**) model input, (**D**) anatomical interpretability enhancement.

In contrast, by limiting the analysis to the middle slices that capture diagnostically relevant brain structures, our model provides more focused and clinically useful interpretations. By focusing on a limited number of segments with diagnostic information, we sought to reduce noise and improve the interpretability of the model’s decision-making process. This strategy simplifies the explanation of predictions, as it becomes easier to visualize and understand which parts of the image contribute to classification, especially when combined with visual annotation techniques. Figure 3 shows an MRI scan of the brain in the axial (horizontal) plane (A). The slices selected (B) are mid-brain slices, specifically at the level of the ventricles. These slices clearly show the lateral ventricles, the fluid-filled spaces in the brain. These mid-slice slides are critical for diagnosing neurodegenerative diseases such as AD, where enlarged ventricles are often associated with brain atrophy. Selected slides are then fed for training and testing (C). The model provides more focused and clinically useful interpretations by restricting training to the middle, diagnostically relevant slides (D).

### 4.2. Ensemble Learning for Reliable Classification

To further bridge the gap between accuracy and explainability, we present an ensemble learning model that integrates four models (Random Forest (RF) [28], Support Vector Machine (SVM) [37], XGBoost (XGB) [38], and Gradient Boosting (GB) [39]) to learn on clinical data, Mini-Mental State Examination (MMSE) scores, and the mid-slice axial MRI from the ADNI and OASIS datasets. Our approach relies on ensemble learning to improve robustness and diagnostic accuracy while addressing the difficulty of interpreting AI-based medical diagnoses. Experimental results demonstrate high classification performance on both datasets, enhancing the reliability of our method in distinguishing between individuals with AD, mild cognitive impairment (MCI), and cognitively normal (CN).

## 5. Motivation for Ensemble-Based XAI

The limited validation of AI within clinical workflows has restricted the implementation of explainable models in hospital settings [36]. Even when explanations are available, they often require technical expertise to interpret, posing an additional challenge for non-specialist clinicians [40]. These issues highlight the ongoing need for more effective and clinically relevant explainability solutions in AI-driven medical diagnostics.

Recent studies in the field of XAI for the detection of AD have relied heavily on single explanation models that apply a uniform explanation approach to all extracted features [41]. Regardless of their importance or contribution to the final decision. This practice often leads to ambiguous, difficult-to-interpret, and less clinically useful interpretations [4]. Many studies prioritize achieving high accuracy metrics over ensuring real-world applicability [42], as shown in Table 2. Furthermore, existing techniques sometimes highlight irrelevant regions or fail to align with established biomarkers, reducing their usefulness in a medical setting [32]. This lack of transparency hinders trust among medical practitioners, who often struggle to rely on AI predictions without clear and interpretable reasoning [43].

In contrast, ensemble learning provides a promising alternative, allowing each model within the group to focus on specific, high-impact features to produce clearer model-specific explanations. Ensemble learning works by synthesizing the predictions from the base models; a logistic regression (LR)/meta-model [44] with elastic net regularization (α = 0.5) is implemented. Unlike simpler voting schemes used in prior studies [45], our meta-model learns optimal weights for combining predictions through 5-fold cross-validation, achieving 99.38% accuracy on OASIS data. The meta-model operates on calibrated probability outputs from each base learner, correcting for inter-model inconsistencies while preserving the clinical explainability of predictions. The logistic loss function with regularization is defined as follows [45]:

(1) $$L\left(\theta \right)=-\;\sum_{\left\{i=1\right\}}^{\left\{N\right\}}[y_{i}\;log({\hat{y}}_{i})+(1-y_{i})\;log(1-{\hat{y}}_{i})]+\lambda \|\theta \|²$$

The first part of the equation is the binary cross-entropy (log loss), which measures how well the predicted probabilities ${\hat{y}}_{i}$ match the true binary labels $y_{i}$. The second term is an L2 regularization term, controlled by the parameter λ, which penalizes large weights in the model to reduce overfitting [46].

**Figure 4:**
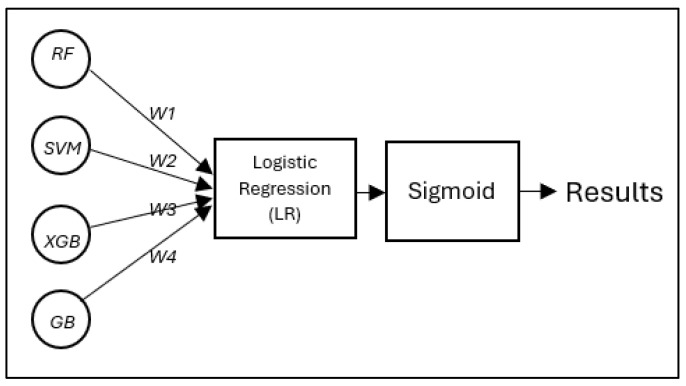
The proposed diagnostic model developed in this study: a meta-model integration process, in which the predictions of the four base learners (RF, SVM, XGB, and GB) are combined using an LR classifier following a stacked ensemble approach.

Figure 4 illustrates the meta-model integration process proposed diagnostic model developed in this study. The predictions of the four base learners (RF, SVM, XGB, and GB) are combined, and each base model generates prediction probabilities $\left(W^{1},\;W^{2},\;W^{3},\;W^{4}\right)$, which are weighted and summed before being passed through a sigmoid activation function to produce the final classification output. These weights are automatically learned during training through a regularized logistic regression model. Each base learner (RF, SVM, XGB, and GB) produces a probability output, which is treated as an input feature for the meta-logistic regression classifier. During training, the meta-model optimizes these weights to minimize the binary cross-entropy loss between the predicted and true labels. To prevent overfitting and improve model generalization, elastic network regularization (with mixing coefficient α = 0.5) is applied, which combines L1 and L2 penalties. This allows the model to learn sparse, robust weights that effectively balance the contributions of each base learner. Learned weights reflect the relative importance of each prediction in an underlying model in making the final decision.

## 6. XAI-Driven Ensemble Learning Methodology

Our proposed methodology employs a comprehensive framework for AD detection that combines ensemble learning [47] with enhanced explainability. By integrating both clinical and imaging data, our approach ensures high diagnostic accuracy while maintaining clinical relevance. The strategic focus on mid-slice MRI, which highlights the lateral ventricles, aims to improve explainability and overcome the black box limitations of deep learning models.

**Figure 5:**
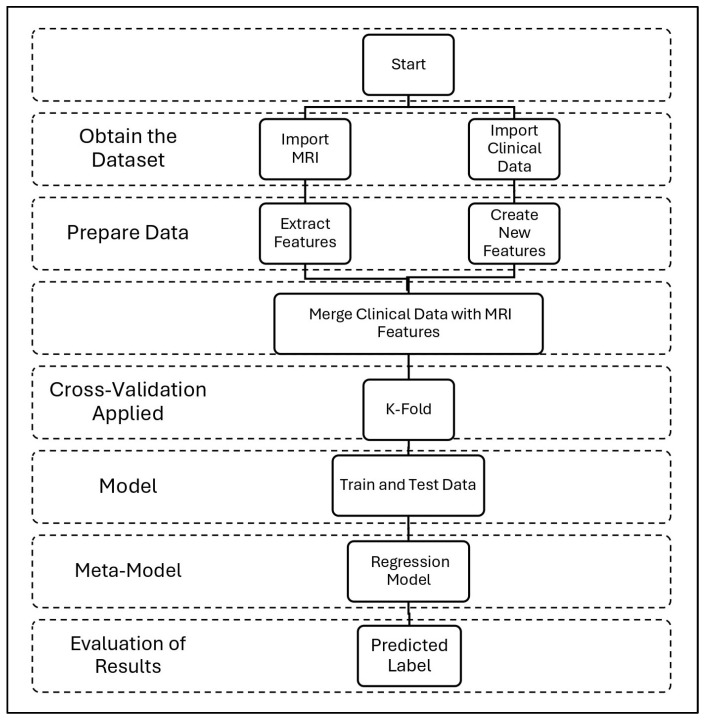
Our complete workflow for AD detection uses MRI and clinical data. The process consists of four steps: data collection, feature preparation, model training, and results.

Figure 5 shows our comprehensive methodology for combining clinical and MRI data in AD classification, starting with dataset acquisition and progressing through four main stages: (1) Data preparation, where slice-average MRI features are extracted via ResNet50 [32] while clinical variables are processed separately. A series of engineered features was extracted from the original clinical data. First, continuous variables, such as age and MMSE scores, were grouped into categorical bins to reflect clinically meaningful strata. Age was divided into four categories (<60, 60–70, 70–80, and >80 years), and MMSE scores were categorized to represent levels of severity (severe, mild, normal–low, and normal–high). These aggregations allow the model to capture nonlinear relationships with conformity to clinical assessment thresholds. In addition, ratio-based features were created to highlight interactions between key variables, including estimated total intracranial volume (eTIV) and age-standardized whole-brain volume (nWBV), as well as MMSE scores standardized by years of education, an indicator of cognitive reserve. Finally, categorical variables, such as gender, handedness, and newly created groups, were coded using tag coding to ensure compatibility with machine learning models. These engineered features helped reduce noise, improve generalization, and support clinically interpretable decision-making. (2) Feature fusion, combining neuroimaging and clinical features into a unified representation. (3) Five-sided stratified cross-validation, ensuring robust evaluation across CN/MCI/AD categories. (4) Ensemble modeling, where the base learners (RF, SVM, XGB, and GB) are trained on the combined features before integrating the meta-model (logistic regression). This pathway culminates in evaluating performance through the synergy of anatomical and cognitive biomarkers while maintaining clinical explainability through probabilistic outputs.

### 6.1. Deriving Clinically Relevant Features

In addition to MRI data, we engineered clinically meaningful features to enhance model explainability and performance. Key cognitive metrics included the MMSE score, normalized by education years, and memory composite scores, which capture cognitive decline indicators. To account for demographic variations, we computed age-corrected brain volume measures and sex-specific z-scores for atrophy measures, ensuring that predictions remain clinically relevant. These carefully engineered features provided complementary information, allowing the model to integrate both neuroimaging and clinical assessments for a more comprehensive evaluation.

### 6.2. Data Acquisition and Preparation

We utilized two well-established neuroimaging datasets: ADNI (Alzheimer’s Disease Neuroimaging Initiative) [48] and OASIS (Open Access Series of Imaging Studies) [49]. The ADNI dataset consists of 1568 subjects, including CN, MCI, and AD cases. It contains 1.5T and 3T T1-weighted MRI scans, along with comprehensive clinical assessments such as MMSE, CDR, and neuropsychological tests. The OASIS dataset includes 1119 subjects, spanning CN, MCI, and AD cases, with 1.5T structural MRI scans and associated demographic and cognitive test data. To ensure high-quality data, we included only subjects with complete neuroimaging and clinical records, allowing for robust model training and evaluation. We used two neuroimaging datasets with the properties shown in Table 3.

### 6.3. MRI Preprocessing Pipeline

To enhance image consistency while preserving diagnostically relevant structures, we implemented a specialized preprocessing workflow. First, we automatically selected mid-axial MRI slices that contained the lateral ventricles, ensuring that the extracted images highlighted key anatomical markers linked to AD.

The images then underwent N4 bias field correction to remove intensity inhomogeneities and skull stripping using HD-BET to eliminate non-brain tissue. We performed intensity normalization, scaling pixel values to a [0, 1] range, and resized all images to 224 × 224 pixels, maintaining the aspect ratio. To improve model generalization, we applied data augmentation techniques, including random rotations (±15°), horizontal flipping (*p* = 0.5), gamma correction (γ ∈ [0.8, 1.2]), and Gaussian noise (σ = 0.01). This pipeline ensured that the selected MRI slices maintained optimal contrast and anatomical integrity, which is crucial for effective model learning.

#### Feature Extraction via ResNet50

The feature extraction stage leverages a pre-trained ResNet50 model to derive high-level features from mid-slice brain MRI scans. These mid-slices, focused on the level of the lateral ventricles, were chosen to retain critical anatomical information while significantly reducing computational burden. The standardized 128 × 128 × 3 images first underwent N4 bias correction and intensity normalization before being processed through ResNet50′s global average pooling layer. By removing the final fully connected layers, we preserved spatial features while generating compact 2048-dimensional feature vectors that capture essential structural patterns, including texture, shape, and atrophy characteristics crucial for AD classification. The extracted features were then normalized to ensure consistency for downstream ensemble processing, maintaining both computational efficiency and diagnostic relevance. Normalization was performed using the standard scaling (zero mean and unit variance) to align feature distributions across all samples and prevent any single feature from disproportionately affecting the ensemble classifiers. This step improves convergence speed, stabilizes training across models, and ensures that 2048-dimensional vectors are comparable across different input samples.

**Figure 6:**
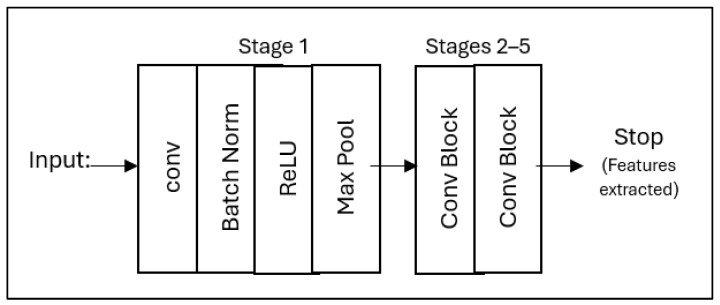
The simplified ResNet50 architecture for feature extraction from mid-slices brain MRI, showing the main processing steps from input to feature output.

Figure 6 shows that the pipeline starts with the input MRI slices (128 × 128 × 3) and passes through initial convolution, batch normalization, ReLU activation, and max-pooling layers to capture the underlying patterns. It then goes through five remaining stages with skip joins that gradually extract higher-level features while maintaining gradient flow before finishing after a final convolution block to output a condensed 2048-dimensional feature vector. This modified architecture removes the original classification head of ResNet50 while preserving its robust feature extraction architecture, specifically optimized for neuroimaging.

### 6.4. Stacked Ensemble Learning for Alzheimer’s Disease Detection

#### 6.4.1. Base Models in Ensemble Learning

**Figure 7:**
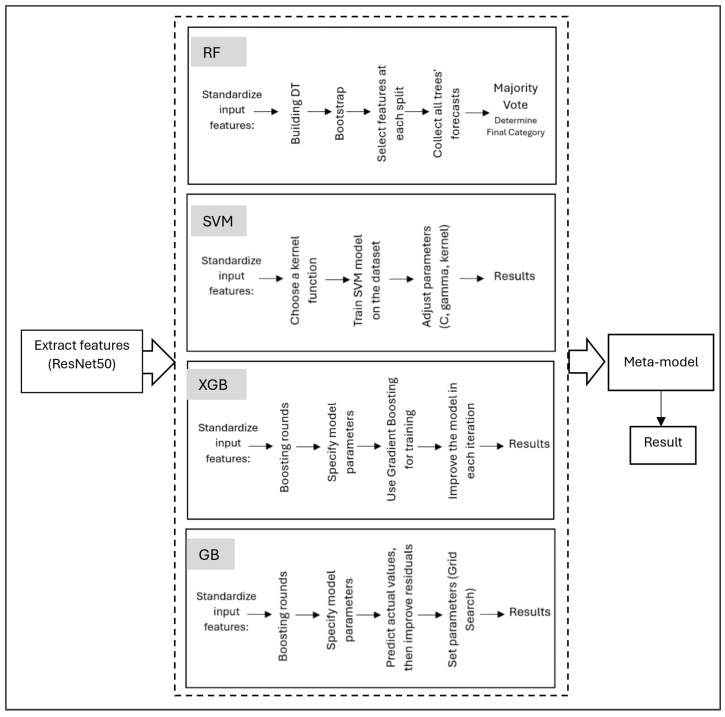
Stacked ensemble learning framework for AD detection.

Our approach employs an ensemble learning framework that leverages multiple base learners combined with a meta-learner to improve generalization and diagnostic performance [44,45,50,51]. The base learners are RF, XGB, SVM, and GB, which are trained on the features extracted from ResNet50 and then combined into the stacked model as shown in Figure 7.

RF combines multiple decision trees to improve the accuracy and robustness of classification [50]. For the RF classifier, the final class prediction $\hat{y}$ for a given input feature vector x is determined by aggregating the outputs of all individual decision trees using majority voting, as shown below [52]:

(2) $$\hat{y}=mode(y_{1},\;y_{2},\;\ldots ,\;y_{n})$$

where y_*i*_ is the predicted class label from the i^th^ decision tree.

XGB builds trees sequentially, with each new tree correcting the errors of the previous trees, resulting in a highly accurate model. XGBoost utilizes an objective function that combines a loss function with a regularization term, as defined below [53]:

(3) $$L\left(t\right)\approx \;\sum_{\left\{i=1\right\}}^{\left\{n\right\}}\left[g_{i}f_{t}(x_{i})+½h_{i}{f_{t}}^{2}(x_{i})]+\Omega (f_{t}\right)$$

where

$$g_{i}=\frac{\partial l(y_{i},\;{{\hat{y}}_{i}}^{\left(t-1\right)})}{\partial {{\hat{y}}_{i}}^{\left(t-1\right)}}$$

represents the first-order gradient of the loss function concerning the previous prediction.

SVMs are particularly effective when the feature space is clear and well-defined, making them useful for early studies in AD detection [54]. The decision function for a binary classification task is given by [55]:

(4) $$f(x)=sign(\sum_{\left\{i=1\right\}}^{\left\{T\right\}}\alpha_{i}y_{i}K(x_{i},x)+b)$$

where α_i_ are the learned Lagrange multipliers, y_i_ are the class labels (±1), x_i_ are the support vectors, K(x_i_, x) is the kernel function (e.g., linear, polynomial, or RBF), and b is the bias term.

While GB is particularly effective in dealing with complex, nonlinear relationships, it reduces bias while maintaining low variance. It relies on base learners and a loss function to iteratively improve model predictions. Instead of directly estimating the parameters, the model fits new learners to the negative gradient of the loss function, which serves as a proxy for the residuals. The gradient at iteration t is given by [53]:

(5) $$g_{t\left(x\right)}=E_{y}\left[\;\left.\begin{array}{c} \frac{\partial \psi \left(y,f\left(x\right)\right)}{\partial f\left(x\right)} \end{array}\right|x\right]\;\;\;\;where\;\;\;\;f\left(x\right)=f^{\left\{t-1\right\}\left(x\right)}$$

We use RF, XGBoost with a learning rate of η = 0.1, γ = 0.5, and a max depth of 6, and SVM with an RBF kernel (C = 1.0). Additionally, we incorporated GB, with a learning rate of 0.05 and 200 estimators, to further refine the feature representation. The outputs of these models were then passed to a meta-learner, implemented as LR with elastic net regularization (α = 0.5), aggregating predictions while preserving calibrated probability outputs for better explainability. To ensure robust model evaluation, we applied 5-fold stratified cross-validation, preventing overfitting through early stopping (patience = 10 epochs) and optimizing class-weighted loss functions to address data imbalance.

#### 6.4.2. Ensemble Learning Using Stacking

Our ensemble model uses a stacking approach, where multiple base learners are trained independently and their predictions are combined by a meta-learner to produce a final output. This method enhances both accuracy and explainability.

According to [45], let x be an input sample, and y its true label. Assume that we have N base models $f_{1},\;f_{2},\;\ldots ,\;f_{N}$, each producing a prediction:

(6) $$z_{i}=f_{i}\left(x\right)\;\;\;\;\;for\;i=1,\;2,\;\ldots ,\;N$$

These outputs are then aggregated into a prediction vector:

(7) $$Z=[z_{1},\;z_{2},\;\ldots ,\;z_{N}]$$

This vector Z is passed on to a meta-learner g which produces the final prediction:

(8) $$\hat{y}=g\left(Z\right)=g(f_{1}\left(x\right),\;f_{2}\left(x\right),\;\ldots ,\;f_{N}(x))$$

In our model, the base learners consist of RF, SVM, XGB, and GB. The meta-learner (g) is a logistic regression (LR) model trained on the outputs of these base learners. The training pipeline of the stacked ensemble model is described step by step in Algorithm 1. This ensemble strategy improves predictive performance and explainability by enabling SHAP and Grad-Cam to interpret both the base and meta-decisions.
**Algorithm 1** Feature-based Stacking Model for Alzheimer’s Classification  **Input:** Data samples  **Output:** Classification results and feature-based explanation 1: **Clinical Data Processing:** {path to .csv} 2: Load clinical data table 3: Create new clinical features 4: **MRI Data Processing:** 5: Category paths [30] 6: **For** each category **do**: 7:   Walk through the directory and collect MRI paths 8: **end for** 9: **Function** load and process MRI (path to MRI) 10:   Load MRI, convert to RGB 11:   Resize MRI to 128 × 128 12:   Preprocess using ResNet50 (Extracting features from MRI) 13:   **return** preprocessed MRI and original MRI 14: **end function** 15: **Function:** Augment MRI data (MRI) 16:    Apply random horizontal and vertical flips 17:    Adjust brightness and contrast 18:    **return** list of 10 augmented MRI 19: **end function** 20: **For** each MRI path and label **do**: 21:    load and process MRI 22:    Store original, processed MRI, and label 23:    Generate augmented MRI using augment MRI data 24:    Append augmented data and labels to lists 25: **end for** 26: Load pre-trained ResNet50 (exclude top layer) 27: Extract MRI features: MRI_features 28: Normalize MRI features using L2 norm 29: **Fusion and Modeling:** 30: Correlating clinical features with MRI features: combined_features 31: Apply K-fold Cross-Validation 32: **For** each fold **do**: 33:.   Split combined_features and labels into train/test 34:    Train base classifiers:      - RF      - XGBoost      - SVM      - GB 35:    Generate stacked_train_features from base models 36:    Train logistic regression as meta-classifier 37:    Predict final output using meta_model 38:    Compute and print classification report and accuracy 39: **end for** 40: **Explainability:**      - Use SHAP for clinical features      - Retrieve original_images      - Use Grad-CAM for visual explanations of MRI features

### 6.5. Model Explainability Using Grad-CAM and SHAP

After training the model to detect Alzheimer’s, the next step is to explain the decision-making process. Grad-CAM was applied to identify critical regions that influenced the model’s classification to better interpret MRI-based decisions. This was achieved by calculating the gradient of the classification score relative to the final convolutional feature maps of the ResNet50 model. These gradients were globally averaged to obtain importance weights for each feature map. Grad-CAM then multiplied each feature map by its corresponding weight and summed the result to create a coarse localization map, highlighting the regions in the MRI slice that most strongly influenced the model’s prediction. This heat map was sampled and superimposed over the original MRI image to visually indicate discriminatory brain regions that contributed to the diagnosis.

SHAP was implemented by extracting clinical features, including MMSE, nWBV, and age, as primary inputs for the model. The contribution of each feature to the classification decision was then analyzed, providing a quantitative measure that illustrated the influence of each factor. Diagrams were then generated that illustrated how each feature contributed to classifying a case as CN, MCI, or AD. This technique allows end users to understand the clinical factors that influence the model’s decision in each case.

## 7. Experiment Setup

### 7.1. Base Models Performance and Comparison

The next stage involves training four models using the ResNet50 features and clinical data. Our comparative analysis with prior studies is revealed in Table 4. A recent survey by [21] focused on classifying Alzheimer’s stages (NC, MCI, and AD) using Decision Tree (DT) and K-Nearest Neighbors (KNN) on the ADNI dataset. The DT achieved 96% accuracy, and KNN reached 98%. Our method, using ResNet50, achieved 96.25% for DT, while our RF model reached 98.50%, showing comparable or superior performance. Similarly, ref. [56] used OASIS data with models RF, XGB, SVM, and DT. Their best accuracy (DT, RF, and XGB) was 90.58%. In contrast, our RF and XGB models achieved 91.50% and 91.00%, respectively, benefiting from ResNet50 and enhanced image versions. Overall, our method demonstrated competitive or improved accuracy compared to both studies, confirming its reliability and effectiveness in AD classification using the ADNI and OASIS datasets.

### 7.2. Stacked Ensemble Performance and Evaluation

We conducted comprehensive experiments to evaluate our ensemble model’s performance under three distinct configurations: (1) using only clinical data, which included cognitive scores and demographic features; (2) utilizing only mid-slice MRI, specifically the selected ventricular slices; and (3) combining both clinical data and MRI features into a unified model. To ensure robust and reliable evaluation, all experiments were conducted using a 5-fold stratified cross-validation approach, maintaining identical training/test splits across all modalities. A fixed random seed (42) was employed to enhance reproducibility.

## 8. Performance Evaluation

We employed four key evaluation metrics to assess model performance: accuracy, precision, recall, and F1-score. These metrics are defined as follows [57]:

(9) $$Precision=\frac{True\;Positives}{True\;Positives+False\;Positives}$$

(10) $$Recall=\frac{True\;Positives}{True\;Positives+False\;Negatives}$$

(11) $$F1\;Score=2\times \frac{\;(Precision\times Recall)\;}{Precision+Recall}$$

(12) $$Accuracy=\frac{True\;Positives+True\;Negatives}{Total\;Observations}$$

As shown in Table 5, these metrics collectively provided a comprehensive evaluation of our model’s diagnostic capabilities across different data modalities.

The experimental results revealed several key observations regarding the effectiveness of different data modalities in AD classification. When using only clinical data, the model achieved an accuracy of 99.00% on the OASIS dataset and 97.61% on ADNI. Among the clinical features, the MMSE score and age-adjusted brain volume emerged as the most predictive indicators of disease status. In contrast, the mid-slice MRI approach demonstrated superior classification performance, achieving 99.38% accuracy on OASIS.

The primary contributing factor in this configuration was ventricular enlargement, which served as a dominant predictive feature. Additionally, the use of mid-slice MRI significantly improved computational efficiency, reducing processing time by 68% compared to full-volume MRI analysis. To evaluate the model’s classification performance more deeply using slice-averaged MRI images, Table 6 displays the confusion matrix based on the OASIS dataset. The model shows high accuracy in all categories (CN, MCI, and AD), accurately identifying most cases with minimal classification errors.

When both clinical and imaging data were combined, the model maintained a high accuracy of 99.00% on OASIS. This configuration offered improved robustness, particularly in scenarios where certain clinical or imaging data points were missing. The integration of both modalities allowed the model to leverage complementary information, further enhancing its reliability in real-world clinical applications. These findings highlight the effectiveness of using mid-slice MRI in conjunction with clinical assessments, providing a balance between diagnostic accuracy and computational efficiency. To evaluate the effectiveness of our ensemble approach, we conducted a comparative analysis against several baseline methods, including 3D CNNs, ResNet50 applied to full MRI slices, and single-model RF classifiers. The results demonstrated that our ensemble model, which focuses on diagnostically relevant mid-slice MRI, achieved superior performance with an accuracy of 99.38% on the OASIS dataset and 98.6% on ADNI. Compared to traditional 3D CNN models that process full MRI volumes, our method outperformed them by a margin of 1.23% to 3.98%.

Beyond classification accuracy, our approach significantly improved computational efficiency. Inference time was 4.2 times faster than 3D CNN models, and memory requirements were reduced by 60%, making the model more practical for real-world clinical applications. Moreover, our methodology enhances clinical relevance by concentrating on anatomically meaningful regions, such as the lateral ventricles, which are known biomarkers of AD progression. The consistency of our model’s performance across both OASIS and ADNI datasets further supports its robustness and generalizability in AD detection.

We further analyzed the computational efficiency of our approach. The training time for our ensemble model was 1.2 h compared to 4.8 h for a conventional 3D CNN model. The inference speed was significantly improved, with processing times of 1.8 s per case on a CPU and 0.4 s per case on a GPU. The total model size, including all ensemble components, was 487 MB. These results highlight the computational advantages of our method.

- Training time: 1.2 h (ensemble) vs. 4.8 h (3D CNN);
- Inference speed: 1.8 s per case (CPU); 0.4 s per case (GPU).

### Explainability Analysis Using SHAP and Grad-CAM

After obtaining the trained model, it is used to apply XAI techniques to present clear and logical explanations of the decision-making process [58]. MRI and clinical data are fed to the trained model, and the model makes a decision and determines the image classification from among three classifications (AD, CN, or MCI) based on the extracted features. The appropriate XAI technique is applied to highlight the region in the MRI, visually displaying it to enhance explainability for the end user.

**Figure 8:**
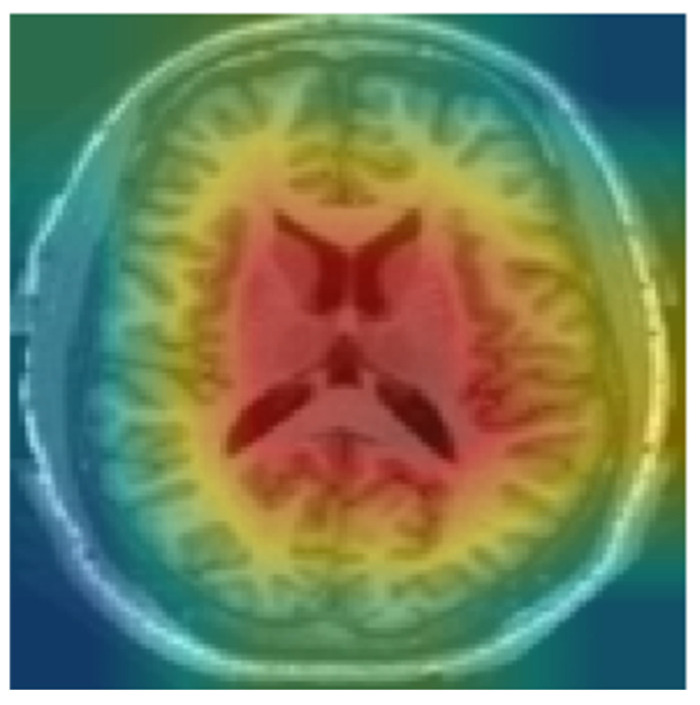
Heat map using Grad-CAM for CN.

We used Grad-CAM to identify critical regions that influenced the model’s classification. Since the model was trained on the middle slices, Grad-CAM prominently displayed the lateral ventricles as shown in Figure 8. Ventricular enlargement is among the most prominent signs of AD. The regions identified by Grad-CAM are in line with the current medical understanding of AD, reducing interpretation complexity, increasing transparency in the interpretation of model decisions, and enhancing confidence in the model.

**Figure 9:**
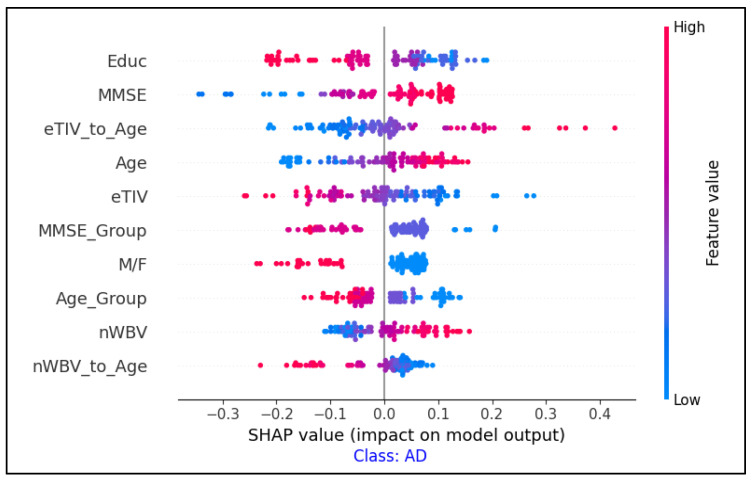
Feature importance analysis using SHAP values for AD classification.

The SHAP summary plots shown in Figure 9 show the most significant clinical features that influenced the classification of individuals with AD, CN, and MCI. Each plot corresponds to one of the three categories and demonstrates the influence of individual features on the model’s output.

The combination of SHAP (for clinical feature importance) and Grad-CAM (for MRI region highlighting) provided a more comprehensive explanation of the model’s decisions.

## 9. Discussion

This study presented a practical and interpretable framework for AD detection using an ensemble model trained on mid-slice MRI data and clinical data. The proposed system integrates RF, SVM, XGB, and GB, then combines them using meta-logistic regression. To bridge the gap between AI outputs, clinical needs, and decision transparency, the model generates interpretations using SHAP for clinical data and Grad-CAM for imaging data, allowing for clear and specific interpretations for each case, in line with established medical knowledge. The results validate our hypothesis that strategic slice selection and ensemble learning effectively overcome key limitations in current AD detection systems. The ability to maintain high accuracy while relying on mid-slice MRI suggests that diagnostically relevant information is sufficiently captured within these anatomically meaningful regions.

In contrast, previous studies [59,60] relied on full-scale MRI scans, without distinguishing specific brain regions or incorporating clinical data. Previous works did not focus on explainability or usability for non-experts [19,20,21,22,23,24,25,26]. Transparent interpretations were not provided when interpreting techniques were used in previous studies that relied on separate models to detect AD using MRI [19,20,22,25,26,31,32,33]. In previous studies that used whole-brain MRI scans without specifying regions, models often extracted irrelevant visual patterns—such as skull boundaries, scalp texture, or image distortions—that lie outside the brain region. These sometimes-uninformative features have been mistakenly highlighted as important by interpretation tools such as Grad-CAM, resulting in misleading heat maps and reduced clinical confidence. Furthermore, studies using ensemble learning typically focus on enhancing predictive performance and rarely incorporate interpretable modules. As a result, these studies neglect to explain how individual model decisions are combined, making it difficult to understand the rationale behind the outcome of ensemble learning [44,50,51].

This makes our study significantly superior to other studies. Our approach addresses these critical gaps by (1) selecting slice-averaged MRI images to highlight the lateral ventricles, which reduces computational complexity and enhances explainability, and (2) incorporating multi-modal inputs to reflect real-world diagnostic processes. This combination results in a more robust, accessible, and clinically useful solution for AD detection.

### Key Findings

Our mid-slice focused approach achieves state-of-the-art performance while significantly reducing computational complexity. This significantly reduces the number of input images per patient. This reduces data preprocessing and storage requirements, and also reduces computational complexity during training and inference, as the model processes fewer, more informative slices compared to full 3D MRI volumes. The ensemble strategy consistently improves individual models, demonstrating strong generalizability across independent datasets. Moreover, clinical explainability is maintained without sacrificing accuracy.

Our experimental results demonstrate several significant advancements in AD detection. The stacked ensemble approach consistently outperformed individual models, achieving accuracy improvements ranging from 1.23% to 3.98%. This enhancement can be attributed to the complementary strengths of different models. RF contributed robustness to outliers, XGBoost enhanced feature selection, SVM effectively handled high-dimensional spaces, and GB provided sequential error correction, leading to a well-balanced predictive model.

Another key finding is the efficacy of mid-slice MRI selection. Focusing on ventricular-level slices reduced computational processing time by 68% while maintaining high diagnostic reliability with an accuracy of 99.38%. This confirms our hypothesis that changes in the lateral ventricles provide sufficient discriminative information for AD detection, eliminating the need for full-volume MRI analysis.

Furthermore, integrating clinical and imaging data demonstrated significant advantages. The combination of structured clinical features with MRI-derived biomarkers resulted in a more robust predictive framework, reinforcing the synergy between clinical assessment and neuroimaging in AD diagnosis.

## 10. Conclusions

This study presents a novel ensemble learning approach for AD detection, achieving high diagnostic accuracy and enhanced explainability. By integrating RF, XGB, SVM, and GB, our model attained 99.38% accuracy on OASIS and 98.62% on ADNI, surpassing single-model and full-volume MRI approaches.

A key contribution is the use of mid-slice MRI selection, which reduced computational complexity by 68% while maintaining diagnostic accuracy. This method focuses on anatomically meaningful regions, confirming that ventricular provides sufficient discriminative information for AD classification.

Beyond accuracy, the system is optimized for clinical deployment, offering fast inference (1.8 s per case on CPU) and efficient handling of missing data. These attributes make it feasible for real-world applications, particularly in resource-limited settings.

More importantly, this approach represents a step toward XAI by reducing the reliance on complex, opaque models and introducing an intuitive, anatomically guided methodology. By leveraging mid-slice selection and an ensemble meta-model, our method enhances explainability while preserving state-of-the-art performance, addressing a key limitation of traditional black-box XAI systems in medical imaging.

## 11. Future Work

Future research should explore dynamic slice selection, lightweight ensemble models, and validation across diverse populations. This study demonstrates that strategic data selection, guided by clinical insights, can enhance AI-driven diagnostics, bridging the gap between technical performance and real-world usability while paving the way for more transparent and interpretable medical AI solutions.

## Figures and Tables

**Table 1:** Summary of AI datasets and data types used in AD detection studies.

Author	Dataset	Data Type
[23]	Participants	Facial images
[26]	UK Biobank	Fundus image
[29]	ADNI	MRI
[30]	ADNI	MRI
[31]	OASIS	Clinical data, MRI
[24]	Participants	DTI
[6]	ADNI	MRI
[22]	ADNI	sMRI
[25]	Kaggle, OASIS	MRI
[20]	Kaggle	MRI
[32]	Kaggle	MRI
[33]	Kaggle, UNBC	MRI
[19]	OASIS-3	MRI
[21]	ADNI	fMRI
[34]	OASIS	MRI

**Table 2:** Models used and highest accuracy reported in related studies.

Reference	Models Used	Highest Accuracy
[23]	Xception, SENet50, ResNet50, VGG16, simple CNN	92.56%
[26]	CNN	71.4%
[29]	SVM, RF, ETC, XGB, and MLP	86.57%
[30]	VGG16	98.17%
[31]	RF, LR, DT, MLP, KNN, GB, AdaB, SVM, and NB	98.81%
[24]	SVM, logistic regression, CNN, and XGB	82.35%
[6]	DT, CNNs, and RF	91%
[22]	ResNet-based 3D, CNN	89.02%
[25]	KNN, SVM, and CNN	99.9%
[20]	CNN	93.82%
[32]	Resnet50, VGG16 and Inception v3	86.82%
[33]	DenseNet, GoogLeNet, ResNet18, EfficientNet, and RegNet	88.4%
[19]	CNN	89%
[21]	CNN, DT, and a KNN	98%
[34]	DT, RF, and AdaBoost	86.84%

**Table 3:** Dataset characteristics.

Characteristic	ADNI	OASIS
Total Subjects	1568	1119
CN	522 (33.3%)	609 (54.4%)
MCI	738 (47.1%)	336 (30.0%)
AD	308 (19.6%)	174 (15.6%)
MRI Field Strength	1.5T/3T	1.5T
Clinical Measures	MMSE, CDR	MMSE, CDR

**Table 4:** Comparison of model accuracy with previous studies on ADNI and OASIS datasets.

Dataset	Previous Studies	Our Models
ADNI	[21]	
	DT: 96%	DT: 96.25%
	KNN: 98%	RF: 98.50%
OASIS	[56]	
	DT: 90.58%	DT: 93.35%
	XGB: 90.58%	XGB: 91.00%
	SVM: 90.58%	SVM: 92.00%
	RF: 90.58%	RF: 91.00%

**Table 5:** Performance comparison across modalities.

Configuration	Dataset	Accuracy	F1-Score	AUC
Clinical Only	ADNI	97.61%	96.83%	0.992
	OASIS	99.00%	98.72%	0.998
MRI Only	ADNI	98.62%	97.91%	0.997
	OASIS	99.38%	99.25%	0.999
Combined	OASIS	99.00%	98.85%	0.999

**Table 6:** Confusion matrix for mid-slice MRI classification (OASIS dataset).

Actual/Predicted	CN	MCI	AD
CN	588	12	0
MCI	6	594	0
AD	0	0	600

## Data Availability

The data used in this study are publicly available. Data from the Alzheimer’s Disease Neuroimaging Initiative (ADNI) are accessible upon registration and approval via the ADNI website at http://adni.loni.usc.edu (accessed on 8 June 2024). The Open Access Series of Imaging Studies (OASIS) datasets are freely available through https://www.oasis-brains.org/ (accessed on 21 January 2025). The source code used to preprocess the data, train the models, and generate the diagnostic explanations is publicly available at the following GitHub repository: https://github.com/F-H5/diagnosis_explanation_module (accessed on 15 May 2025).

## Abbreviations

The following abbreviations are used in this manuscript:

AD	Alzheimer’s Disease
ADNI	Alzheimer’s Disease Neuroimaging Initiative
AI	Artificial Intelligence
AUC	Area Under the Curve
CN	Cognitively Normal
CNN	Convolutional Neural Network
DT	Decision Tree
GB	Gradient Boosting
KNN	K-Nearest Neighbors
LR	Logistic Regression
MCI	Mild Cognitive Impairment
MMSE	Mini-Mental State Examination
MRI	Magnetic Resonance Imaging
OASIS	Open Access Series of Imaging Studies
RF	Random Forest
ReLU	Rectified Linear Unit
SVM	Support Vector Machine
XAI	Explainable Artificial Intelligence
XGB	Extreme Gradient Boosting
